# Training Deep Neural Networks with Novel Metaheuristic Algorithms for Fatigue Crack Growth Prediction in Aluminum Aircraft Alloys

**DOI:** 10.3390/ma15186198

**Published:** 2022-09-06

**Authors:** Muhammad Hamza Zafar, Hassaan Bin Younis, Majad Mansoor, Syed Kumayl Raza Moosavi, Noman Mujeeb Khan, Naureen Akhtar

**Affiliations:** 1 Department of Electrical, Capital University of Science and Technology, Islamabad 44000, Pakistan; 2 School of Electrical and Electronics Engineering, National University of Sciences and Technology, Islamabad 44000, Pakistan; 3 Department of Automation, University of Science and Technology of China, Hefei 230027, China; 4 Department of Engineering Sciences, University of Agder, 4879 Grimstad, Norway

**Keywords:** crack growth rate, artificial intelligence, deep learning, aluminum aircraft alloys, fatigue crack growth prediction

## Abstract

Fatigue cracks are a major defect in metal alloys, and specifically, their study poses defect evaluation challenges in aluminum aircraft alloys. Existing inline inspection tools exhibit measurement uncertainties. The physical-based methods for crack growth prediction utilize stress analysis models and the crack growth model governed by Paris’ law. These models, when utilized for long-term crack growth prediction, yield sub-optimum solutions and pose several technical limitations to the prediction problems. The metaheuristic optimization algorithms in this study have been conducted in accordance with neural networks to accurately forecast the crack growth rates in aluminum alloys. Through experimental data, the performance of the hybrid metaheuristic optimization–neural networks has been tested. A dynamic Levy flight function has been incorporated with a chimp optimization algorithm to accurately train the deep neural network. The performance of the proposed predictive model has been tested using 7055 T7511 and 6013 T651 alloys against four competing techniques. Results show the proposed predictive model achieves lower correlation error, least relative error, mean absolute error, and root mean square error values while shortening the run time by 11.28%. It is evident through experimental study and statistical analysis that the crack length and growth rates are predicted with high fidelity and very high resolution.

## 1. Introduction

There is a significant importance of fracture mechanics in mechanical engineering. Most structural failures in automobiles, aircraft, bridges, and tanks are a result of fatigue crack propagation in materials [1]. Structural failure of a 58-year-old Grumman G73T Turbo Mallard Seaplane is one of the examples that occurred in 2005; the right hand wing of which shed on a domestic flight in the USA. In 2002, a Chinese Boeing 777 crashed mid-air as a result of a structural failure as well. The crash of a Japanese Boeing 747 SR100 was also due to structural failure. Such accidents pose a serious threat to automobile manufacturing companies to survive, as these failures lead towards human loss, as well as enormous economic deficit. This remains to be a matter of serious concern. Crack propagation under fatigue has been comprehensively explored by researchers and professionals in this field and many of them tried to predict it using various methodologies with the help of theoretical and experimental knowledge [2,3].

**Figure 1:**
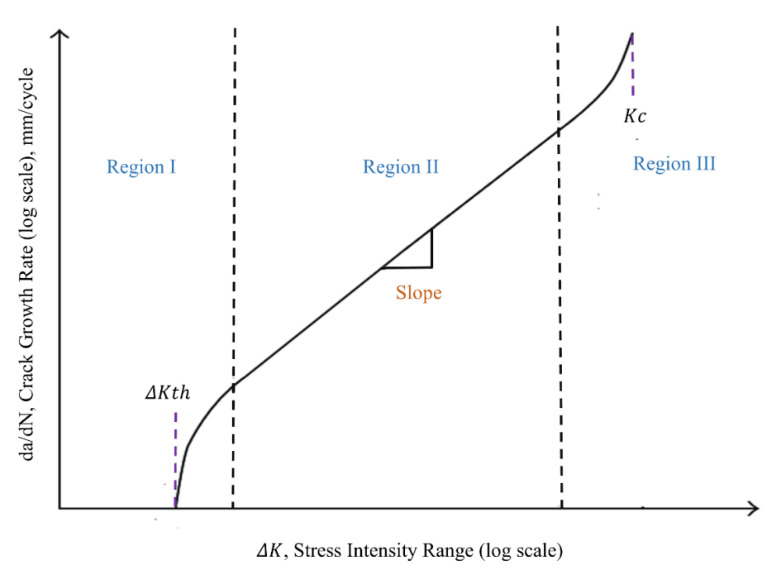
Crack growth rate against stress intensity range.

In engineering, fatigue is a process of material failure at a stage that is far below the actual material strength when subjected to cyclic loading [4]. The traditional models attempt to predict the fatigue crack growth (FCG) rate $\frac{da}{dN}$ with respect to the stress intensity factor (SIF), ΔK [5]. A typical graph is shown in Figure 1. In this figure, a represents the length of the crack, while N represents the number of cycles that leads to failure of structure. The FCG curve is divided into three regions. The crack growth rate is much slower in Region I. In Region II, the graph shows a linear and stable growth followed by a rapid growth in Region III [6]. Paris and Erdogan [7] presented a pioneer model in their study using an extensive pool of data, which is also known as Paris’ law. It presented the accurate modeling of an FCG curve, especially in Region II, which is also called the Paris region. Forman et al. [8] claimed that the stress ratio effect was ignored in the Paris model. The model was improved in their study by considering the stress ratio and crack instability effect. It is stated that this model is more flexible compared to the classical Paris model as it is based upon a large amount of data. Priddle et al. [9] presented a method that improves Paris’ law. However, this model failed to address SIF at threshold, $K_{th}$, average SIF, and stress ratio effects [10]. Kujawski et al. [11] designed a parameter to relate stress ratio and FCG rate, both for long and short cracks in the Paris region. All the above mentioned models cannot handle non-linearity in Region II and III of the graph [12]. A short literature summary has been provided in Table 1.

In comparison to the extensive literature on empirical techniques, machine learning-based models have lesser literature available as this is a relatively modern technique [19]. Jang et al. [20] forecasted the fatigue life of chains used in cranes to lift heavy duty cargo loads using machine learning. Specifically, a logistic regression-based model was used. The experimental data were extracted using strain gauges. Fatigue life prediction in overhead wires was predicted using artificial neural networks (ANNs) by Nowel and Nowel [21]. Yan et al. [22] analyzed the failure of a bridge as a result of an overloading of vehicles using machine learning methodology. This approach gave more accurate results as compared to conventional finite element analysis-based techniques. In [23], the researchers used a radial basis function neural network (RBF-NN) to predict the fatigue crack growth rate in the airplane industry. The problem of vibrations in electrical transmission lines caused by wind, earthquakes, or snow loading was addressed by Pestana et al. using ANNs [24]. Younis [25] carried his work forward by predicting fatigue crack length using radial-based function-NN (RBF-NN). Moreover, optimized neural networks were used to predict the FCG rate in aircraft aluminum structures by Younis et al. [12]. The hill climbing optimized neural network produced better results among the other two by having better relevance with experimental data.

## 2. Fracture Mechanics

**Figure 2:**
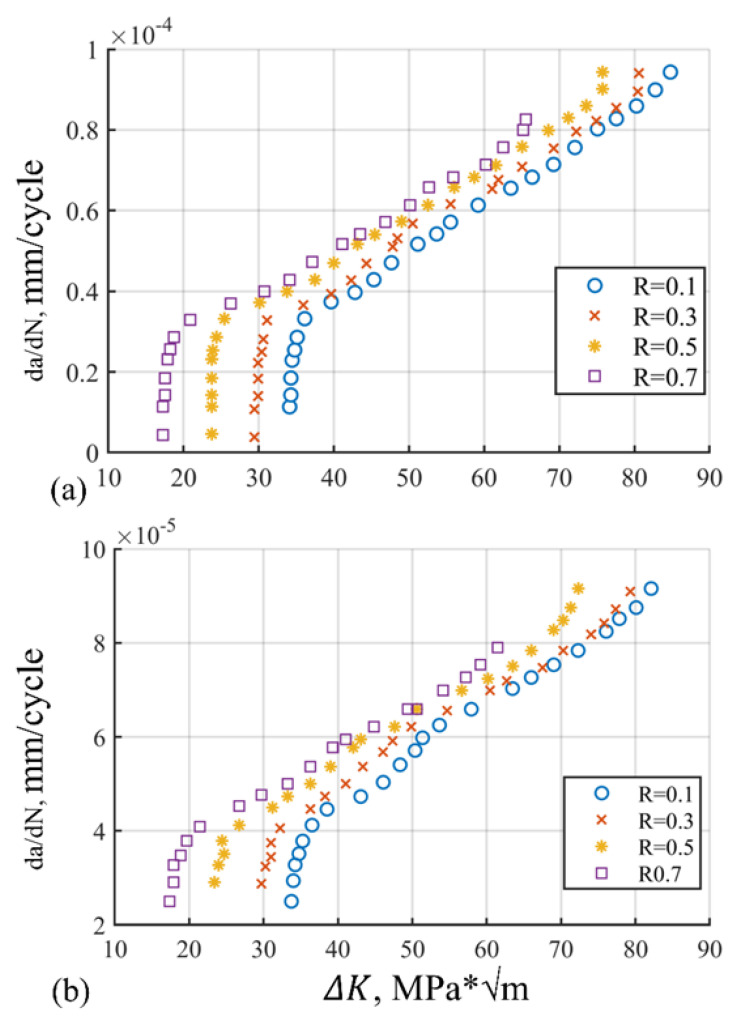
(**a**) Alloy 2324 T39; (**b**) alloy 6013 T651.

Fracture mechanics is the study of crack propagation in materials. All engineering structures are prone to geometrical discontinuities. The size and shapes of such features are crucial as they pose serious concerns about the strength of the component [26]. Structures exposed to cyclic loading fail far below their strength due to fatigue. It refers to the progressive degradation of material due to cyclic loading when subjected to applied force such as stress, strain, and torque [27]. Fatigue crack growth is discussed in context to different crack regions as shown in Figure 2 for 2324 T39 and 6013 T651 materials. Crack initiation is the first phase of crack propagation, followed by stable crack propagation and unstable crack propagation, respectively. In the first region, small cracks initiate that are of the order 10–25 µm or even less. This region is also called the threshold region. In Region II or the Paris region, the crack growth rate is of the order $10^{-6}-10^{-3}\;\mathrm{mm}/\mathrm{cycle}.$ After crossing Region II, the crack propagates exponentially; that is, of the order $10^{3}\;\mathrm{mm}/\mathrm{cycle}$. The Paris region is a main concern of our research, which is explained by Paris and Erdogan [7] in the form of the crack growth equation. It is also called Paris’ law. The stress intensity factor K illustrates load all over the crack tip. The ΔKd can be utilized to correlate the R-ratio effects [27]. Crack propagation is the function of SIF range and ΔK during cycling loading. It is mathematically presented as (1):

(1) $$\frac{da}{dN}=C{\left(\Delta K\right)}^{m}$$

where a is the crack length, dadN is the crack growth rate for load cycle N. C and m are constants that are material dependent. SIF range can be represented as (2):

(2) ΔK=Kmax−Kmin

## 3. Proposed Technique

### 3.1. Dynamic Levy Flight Chimp Optimization Algorithm

In the following section, a new chimp optimization algorithm with Levy Flight random movement (DLFCO) is put forward in order to train DNN. The presented procedure updates the weights and biases of the DNN in order to decrease the cost mapping. DLFCO is an optimization algorithm based on the populace that has the application in the hunting and preying behavior of the chimps to update the weights and biases with the Levy flight random walk in order to obtain global maxima in the exploitation phase. Fusion of LF with the ChoA improves the exploration and exploitation capability of the algorithm [28]. This extremely exploratory attitude enables it to locate the overall best answer, that in turn efficiently trains the DNN model. This trained framework forecasts the fatigue crack growth for the aluminum alloys in aircraft applications. The preceding segment elucidates the chimp optimization and sine–cosine merged chimp optimization algorithm.

#### 3.1.1. Chimp Optimization Algorithm (ChoA)

**Figure 3:**
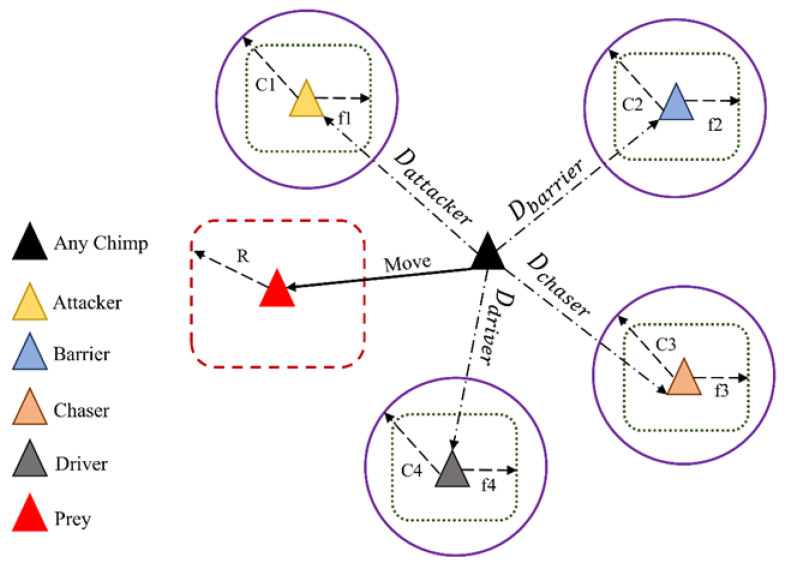
ChoA position updating.

This model obtains its motivation by the intellect of the chimpanzee and breeding actions in cluster hunting. Four techniques are implemented to replicate the attitude, which are attack, chase, barrier, and driver as show in Figure 3. The scientific equations of the driver and attacker of the presented approach are mentioned in (3) and (4), respectively.

(3) $$D=\left|C\cdot \alpha_{prey}-m\cdot \alpha_{chimp}\;\right|$$

(4) $$\alpha_{chimp}\left(n+1\right)=\left|\alpha_{prey}-a\cdot d\right|$$

where *m*, *c*, and *d* are the co-efficient vectors. These factors may be rationalized by (5)–(7).

(5) $$a=2\cdot l\cdot r_{1}-l$$

(6) $$c=2\cdot r_{2}$$

(7) m=chaotic value

where $r_{1}$ and $r_{2}$ represent random numbers in the range of 0–1 and l is a constant which decreases in the direction of a line from 2.5 till 0 all along the iterations and m is a disordered vector. To mathematically simulate this system, the four best results with optimal capability are chosen which are attacker, barrier, chaser, and driver. The remaining inhabitants will upgrade their location with help from the data provided by the best four results. Their statistical demonstration is given by Equations (8), (9), (10), and (11), respectively.

(8) $$d_{attack}=\left|C_{1}\cdot \alpha_{attacker}-m_{1}\cdot x_{n}\right|$$

(9) $$d_{barrier}=\left|C_{2}\cdot \alpha_{barrier}-m_{2}\cdot x_{n}\right|$$

(10) $$d_{chaser}=\left|C_{3}\cdot \alpha_{chaser}-m_{3}\cdot x_{n}\right|$$

(11) $$d_{driver}=\left|C_{4}\cdot \alpha_{driver}-m_{4}\cdot x_{n}\right|$$

The succeeding point of the chimps are restructured consequently using (12)–(15):

(12) $$x_{1}=\alpha_{attacker}-a_{1}\cdot d_{attacker}$$

(13) $$x_{2}=\alpha_{barrier}-a_{2}\cdot d_{barrier}$$

(14) $$x_{3}=\alpha_{chaser}-a_{3}\cdot d_{chaser}$$

(15) $$x_{4}=\alpha_{driver}-a_{4}\cdot d_{driver}$$

Using the above equations, the positions are upgraded through:

(16) $$x_{1}=\frac{x_{1}+x_{2}+x_{3}+x_{4}}{4}$$

Subsequently, after upgrading the positions, (17) is applied.

(17) $$\alpha_{chimp}\left(n+1\right)=\left\{\begin{array}{cc} \alpha_{prey}-x\cdot d & ,\emptyset <0.5\\ chaotic & ,\emptyset <0.5 \end{array}\right.$$

#### 3.1.2. Levy Flight (LF)

**Figure 4:**
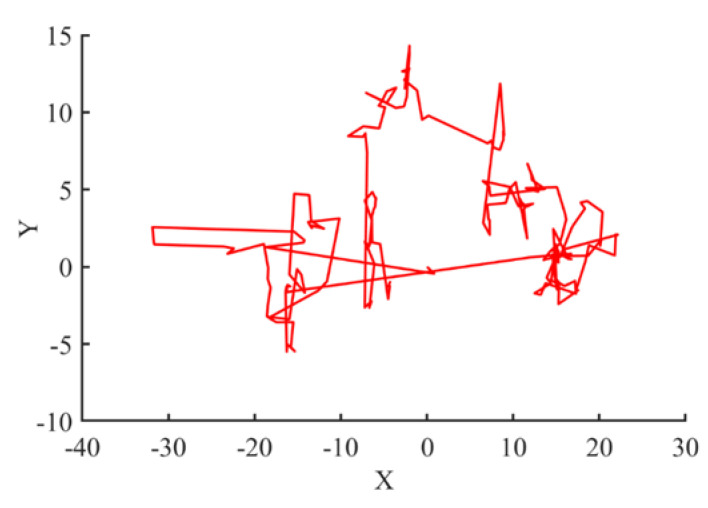
Levy flight movement.

Levy flight is the random walk used for position updating in a search of global maximum position in the area of search. LF is a non-Gaussian distribution based on arbitrary numbers. The Levy flight random walk is presented in Figure 4. The Gaussian method is a relatively stable procedure, containing summation of the Gaussian variables and Gaussian spread. The probability density function is shown in (18).

(18) $$L\left(x\right)=\frac{1}{\pi }\int_{0}^{}e^{-\gamma p\alpha }\cos\left(px\right)dp$$

The Levy spread has two constraints, i.e., α, γ, and is proportional as well, regarding x=0. Where γ is the scaling aspect, γ > 0 and α is between [0, 2] [29]. The factor α defines the spreading figure to attain a number of probability distribution characters; specifically, the end of dispersion is governed by the factor α. Figure 4 provides the chaotic map of the Levy flight showing long and short jumps in the search space.

#### 3.1.3. Improved ChoA with LF

**Figure 5:**
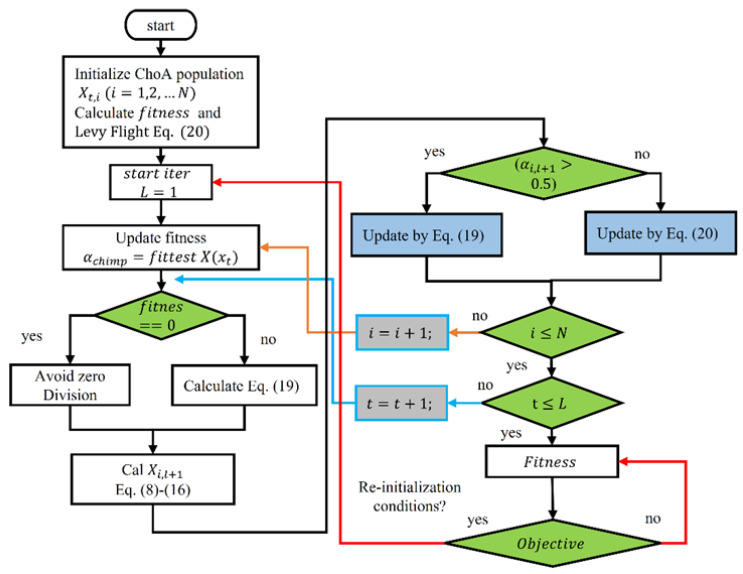
Pseudocode of DLFCO.

The established ChoA keeps its agents posted in the direction of its prey, i.e., the best position on the basis of the driver, chaser, barrier, and attacker positions. On the other hand, the search neurons of the algorithm can become stuck in local minima in some circumstances. So, the premature conjunction issue may pose a threat. In a few circumstances, the acknowledged ChoA is unable to perform the transition from the exploration to exploitation phase and back smoothly. In this section, LF is applied to speak about the above stated limitations. The DLFCO offers more deep searching arrays that lead towards an efficient global search. This arrangement can solve immobility issues as well. In addition, the agent features must be upgraded in DLFCO through all iterations. The chimp’s location can be updated using (19) and (20).

(19) $$x_{chimp}=\frac{x_{1}+x_{2}+x_{3}+x_{4}}{4}+rand\left(0\right)⊕\;Levy\left(W1\right)\;\mathrm{if}\;|a|\;<\;0.5$$

(20) $$\;x_{chimp}=\frac{x_{1}+x_{2}+x_{3}+x_{4}}{4}+rand\left(0\right)⊕\;Levy\left(W2\right)\;\mathrm{if}\;|a|\;>\;0.5$$

where ⊕ designates multiplication in the hierarchical entry, *W*1 and *W*2 are decreasing over the iterations, in that order; these equations are defined in a manner that *W*1 shows a descending angle and *W*2 shows an ascending angle, and both of the parameters show an arbitrary performance. The pseudo code of the proposed technique is presented in Figure 5.

### 3.2. Deep Neural Network (DNN)

An artificial neural network (ANN) is a brain-like structure in which learning is undertaken by experience. It consists of three layers, i.e., input layer, hidden layer, and output layer. The input layer acts as a channel to pass information to the hidden layer by multiplying with weights $W_{ij}$, which are weights from the input layer to the hidden layer. The relationship of input and output data of the hidden layer is presented in Equation (22). The hidden layer applies to the predefined mathematical model which is called the activation function. The output of the hidden layer is passed onto the output layer after multiplying it with weights $W_{jk}$. The relationship between the output and hidden layer is presented by Equation (23).

The number of weights and biases are directly proportional to the number of neurons in the hidden layer. The selection of the number of neurons in the hidden layer depend upon the non-linearity of the dataset. A high number of neurons could result in overfitting, while a lower number of neurons could lead to inefficient training of the ANN. Therefore, in order to deal with a highly non-linear dataset, multiple hidden layers need to be added between the input layer and output layer, which is called the deep neural network.

**Figure 6:**
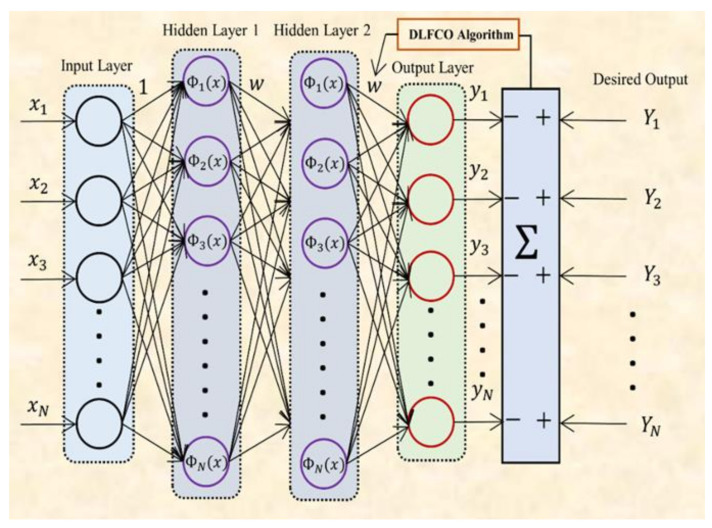
Structure of DNN.

The deep neural network (DNN) is a multi-layered structure like the ANN, but it consists of more than one hidden layer. In this work, a layered DNN is implemented which contains one input layer, two hidden layer neurons, and one output layer, as shown in Figure 6. Six neurons are selected in the hidden layer.

### 3.3. Activation Function

For active mapping of inputs and outputs, a function is required in the hidden layer neurons called the activation function. Each hidden layer neuron has the activation function which is chosen on the basis of application. There are different types of activation functions in classification and regression applications. For classification, the sigmoid function is commonly used as in Equation (21).

(21) $${\overset{˘}{a}}_{i}=\frac{1}{1+e^{-x_{i}}}\;\;Sigmoid\;Function$$

As the fatigue crack growth rate is a regression problem and has a highly non-linear nature, the radial basis function neural network is chosen as the activation function, which is given by (22).

(22) vjyj=exp−∑i=1ryij−y^ ij2μRBF

### 3.4. Cost Function

The cost function or fitness function is the function that tells the difference between actual data and predicted data. This is the function which needs to be minimized during training of the ANN by updating the weights and biases. Various types of cost functions are presented in Equations (23)–(26).

(23) $$\mathrm{MAE}=\frac{\sum_{i=0}^{n}\left|y-y^{\prime }\right|}{n}\;\;Mean\;Absolute\;Error$$

(24) $$\mathrm{MSE}=\frac{\sum_{i=0}^{n}{\left(y-y^{\prime }\right)}^{2}}{n}\;\;Mean\;Squared\;Error$$

(25) $$\mathrm{RMSE}=\sqrt{\frac{\sum_{i=0}^{n}{\left(y-y^{\prime }\right)}^{2}}{n}}\;\;Root\;Mean\;Squared\;Error$$

(26) $$\mathrm{NRMSE}=\frac{\mathrm{RMSE}}{X_{obs,max}-X_{obs,min}}\;\;Normalized\;Root\;Mean\;Squared\;Error$$

The normalized root mean square error (NRMSE) is the cost function which is selected for this problem and is presented in Equation (26). The specifications of the DNN used in this network are presented in Table 2.

### 3.5. Neural Network Training Using DLFCO

Updating weights and biases in the neural network is called the training of the neural network. The back propagation (BP) algorithm is one of the simplest algorithms to train neural networks, but it takes lots of iterations for effective training, which makes BPNN expensive computationally. For effective training of weights and biases, the metaheuristic optimization algorithm is used in this work, which calculates the cost function and updates weights and biases according to the mathematical model of the optimization algorithm.

**Figure 7:**
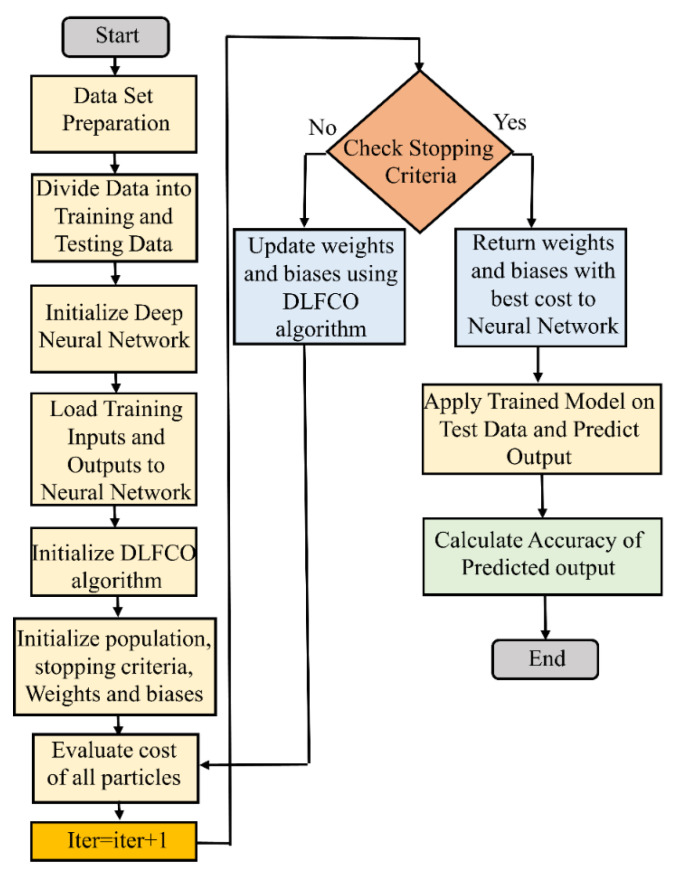
Flow chart for training of DNN using DLFCO.

As shown in Figure 7, firstly, 50 sets of weights and biases are initialized over the search space randomly. These sets of weights and biases are the particle’s position. Then, the DNN structure with two hidden layers having six neurons in each layer are initialized. After that, fitness is calculated for every particle using NMSE, which defines the best particle’s position for the current iteration in the population. After calculation of the best particle, the particle’s position is updated using the optimization algorithm. This phase of optimization is called the exploration phase.

## 4. Results

This section first discusses the data collection for the fatigue crack growth rate, evaluation parameters for the prediction results, and prediction of the growth rate.

### 4.1. Data Collection

The data extraction has been undertaken for dissimilar aircraft structures of aluminum with the range of *R*-ratio from 0.1 to 0.7 after comprehensive experimentation of alloy specimens in the Fracture Technology Associates Laboratory, USA. The experimentation setup includes servo-controlled, hydraulically actuated, closed-loop assembly that is furthermore linked to a standalone PC for information gathering and governing of the factors. Test conditions which are undertaken for experimentation are summarized in Table 3.

### 4.2. Evaluation Parameter

In order to assess diverse techniques, further error directories are applied as well. The grade of spreading in outcomes may be corroborated through normalized root mean square error (NRMSE), given by Equation (27). To specify deviancy of extrapolation, mean absolute error (MAE) and mean absolute percentage error (MAPE) is given by Equations (28) and (29), correspondingly. Furthermore, the relationship between real and forecasted value can be calculated with the help of R-square ($R^{2}$), which is mentioned in Equation (30).

(27) $$\mathrm{NRMSE}=\frac{1}{T}\sqrt{\frac{1}{N}\overset{N}{\underset{i=1}{\sum }}{\left(T_{i}-P_{i}\right)}^{2}}\times 100\%$$

(28) $$\mathrm{MAPE}=\frac{1}{N}\overset{N}{\underset{i=1}{\sum }}\frac{\left|T_{i}-P_{i}\right|}{P_{i}}\times 100\%$$

(29) $$\mathrm{NMAE}=\frac{1}{N}\overset{N}{\underset{i=1}{\sum }}\frac{\left|T_{i}-P_{i}\right|}{P_{i}}$$

(30) $$R^{2}=\frac{\sum_{i=1}^{N}\left(T_{i}-\bar{T_{i}}\right))\cdot {\left(P_{i}-\bar{P_{i}}\right)}^{2}}{\sum_{i=1}^{N}\left(T_{i}-\bar{T_{i}}\right)\cdot \sum_{i=1}^{N}\left(P_{i}-\bar{P_{i}}\right)}$$

where $T_{i}$ represents the actual value, $P_{i}$ is the forecasted value, N is the overall sum of illustrations. $\bar{T_{i}}$ is the mean of the actual output value, $\bar{P_{i}}$ is the mean of forecasted output value.

### 4.3. Prediction Results

**Figure 8:**
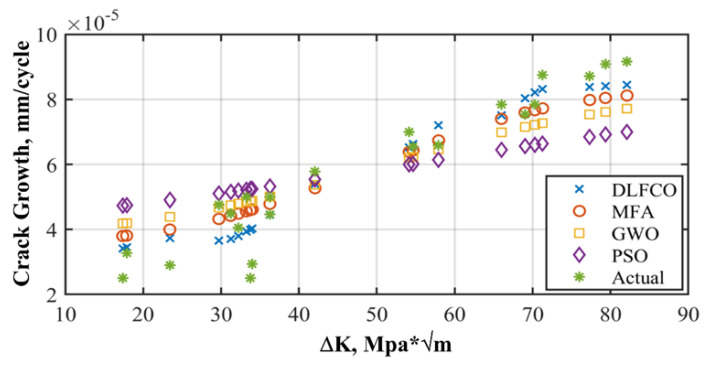
Alloy 7055 T7511 crack growth rate prediction.

**Figure 9:**
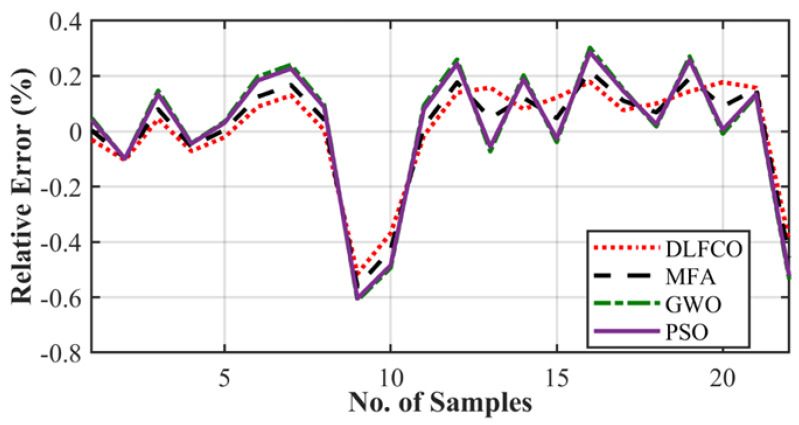
Alloy 7055 T7511 crack growth rate relative error.

**Figure 10:**
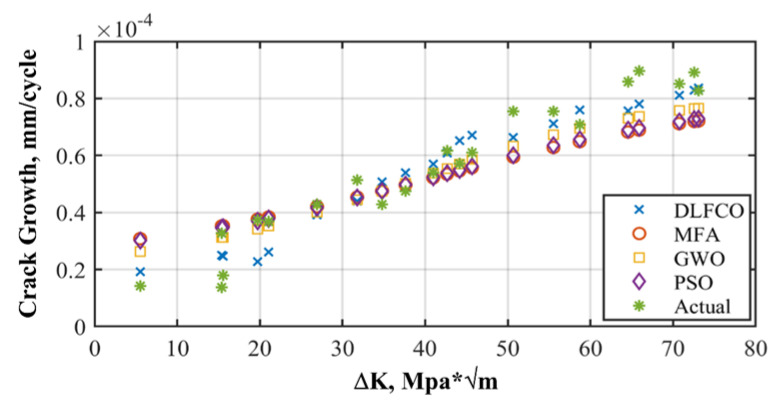
Alloy 6013 T651 crack growth rate prediction.

**Figure 11:**
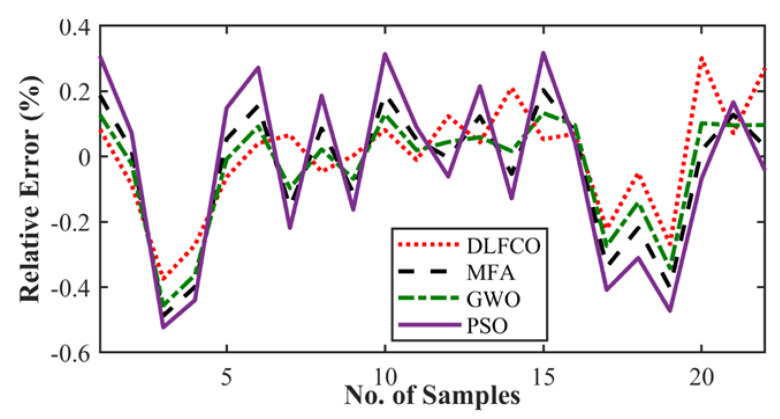
Alloy 6013 T651 crack growth rate relative error.

**Figure 12:**
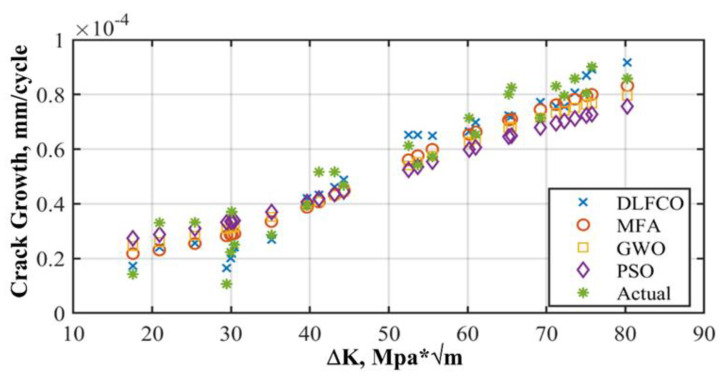
Alloy 2324 T39 crack growth rate prediction.

**Figure 13:**
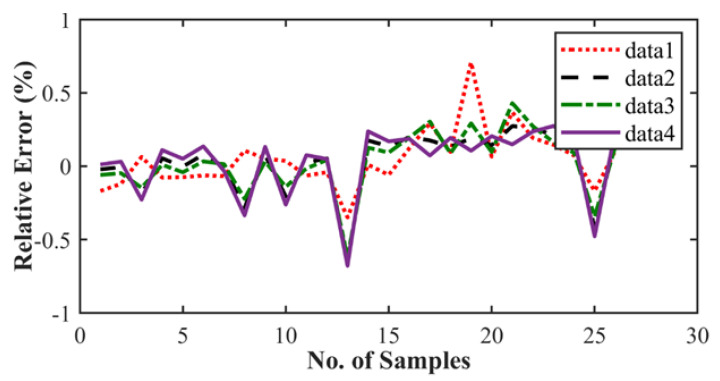
Alloy 2324 T39 crack growth rate relative error.

The results are presented in the graphical, statistical, and qualitative measures. For aircraft alloys, namely, 7055 T7511, 6013 T651, and 2324 T39, prediction results and accuracy of prediction with respect to the sample are compared in Figure 8, Figure 9, Figure 10, Figure 11, Figure 12 and Figure 13 The results show the rate increase as the independent parameters progress non-linearly. The corresponding algorithm performances and statistical analysis is presented in Table 4, Table 5, Table 6, Table 7, Table 8 and Table 9.

## 5. Discussion

To verify the prediction performance of shape and growth rate evolution, the testing set has been employed. The aluminum alloys under experimental data are 6013 T651, 7055 T7511, and 2324 T39. The 6013 T651 is medium strength aerospace alloy that offers enhanced formidability and corrosion resistance. The 2324 T39 alloy exhibits higher strength with better fracture toughness. The 7055 T7511 alloy is predominantly employed in compression-dominated structures due to improvised compressive and tensile strengths. The experiments conducted at the Fracture Technology Associates Laboratory utilize aluminum with an *R*-ratio range of 0.1–0.7 in several experiments. The stress was applied using a servo-controlled, hydraulically actuated, closed-loop assembly equipped with HIL data acquisition and parameter control. Strain gauge transducers measure the displacement that is utilized to measure the length and growth rates secondarily [30].

The performance of the proposed framework has been compared with three highly effective and recently studied optimization algorithms. It includes particle swarm optimization algorithms that provide standard behavior of metaheuristic optimization algorithms, which have the capability to distinguish among sub-optimum solutions. The second algorithm is the grey wolf optimization algorithm (GWO). The improved information sharing mechanism among GWO solutions enhances the accuracy towards optimum solutions. The comparison is made in terms of testing error, training errors, and algorithm run time. A more comprehensive statistical analysis is made for MFA-DNN, GWO-DNN, PSO-DNN, and DLFCO-DNN in Table 8 for statistical indices such as MAE, RMSE, correlation ($R^{2}$), and mean RE [31].

Figure 9, Figure 11 and Figure 13 exhibit the trained DLFCO-DNN estimate RE of predicted values at various samples with high redundancy. The lack of accuracy in competing techniques is primarily associated with over- and/or under-fitting of machine learning algorithms. The stagnation and model fitting issues arise when the predictive values show high variance and low bias due to the noise present in the data. It is avoidable through accurate selection of parameters and preprocessing of data outliers and feature mapping during the pre-processing of the data. Table 4, Table 5, Table 6, Table 7, Table 8 and Table 9 show a summary of the results for da/dN rate prediction using DLFCO-DNN on three aluminum alloys. The testing and training values are compared alongside run time. The best run time has been achieved by a simple criterion that defines the boundary of the small-scale yielding regime, to avoid invalid use of the LEFM parameter, Δ*K*, as the characterizing parameter for the fatigue crack growth rate [32].

While in uniform amplitude loading, the FCG graph—crack growth rate $\left(da/dN\right)$ against SIF (Δ*K*) in log–log measure—normally comprises three portions. Region I denotes the earlier crack propagation. In this region, the crack normally grows in the order of $⩽10^{-6}\;\mathrm{mm}/\mathrm{cycle}$ span. This section of the curve is subjective to the micro-structural appearances, the *R*-ratio (lowest useful stress divided by maximum useful stress), plus the ecological circumstances. In general, it is believed that there subsists a SIF at threshold $\left(\Delta K_{th}\right)$. Under this point, fatigue crack propagation does not take place [33]. Speedy and unsteady fatigue crack growth takes place in Region III of the curve before fracture. A crack progression rate of the order of $\geq \;10^{-3}\;\mathrm{mm}/\mathrm{cycle}$ is ordinary in the case of metals and is asymptotic when crack growth reaches the threshold of fracture toughness $\left(K_{c}\right)$ of the material [34]. This is even though it is mostly dominated by the stress ratio effect, substance micro-cracks, and the specimen width. Moreover, crack growth in the third region of the curve is usually overlooked as a result of trivial leftover fatigue life while arriving in the region. Stable crack growth is observed in Region II of the FCG curve, typically in the range of $10^{-6}\;\;\mathrm{mm}/\mathrm{cycle}\;\mathrm{to}\;10^{-3}\;\;\mathrm{mm}/\mathrm{cycle}$ in the case of metals [35].

It is pertinent to mention that in the case of a hole in the crack route, the crack growth rate in Region I reduces considerably, while in Region II, it elevates [36]. It is noticeable in the trajectory of crack propagation; in a straight line crack progression, KI escalates, but upon the change in direction, KII escalates [37]. The preceding graphs of SIF reveal that all simulated specimen testing data exhibit almost similar numbers for KI and KII throughout crack progression intervals. These insignificant deviations in the final values are dominated by the quantity of phases employed in every package and the mathematical methodology applied. The quantity of phases applied in ANSYS software for model simulation range between 11 and 20, with the total amount of percentage increase organized with the help of software. In FRANC2D/L, the steps are between 32 and 92 with a 0.1 to 0.13 increment per step.

Figure 8, Figure 10 and Figure 12 show comparisons of MFA-DNN, GWO-DNN, PSO-DNN, and DLFCO-DNN fatigue life simulations with experimental outcomes for 6013 T651, 7055 T7511, and 2324 T39. The simulated fatigue crack growth life using the proposed method comes to an impeccable agreement, while the experimental results are provided by Gomes and Miranda [38]. Moreover, the present study outcomes came out to be much more accurate for prediction of fatigue life as linked to the arithmetical results achieved by means of the software. The outcomes of such a study are carefully compared with Quera2D instead of BemCracker2D. Such results, moreover, establish the impact of the opening location regarding the fatigue life of the samples in various areas of impact with ΔK stress intensity range on a log scale [39]. Care must be taken while taking into account the use of ΔK, which is usable for high strength alloys as well [32]. The deterioration caused by the operational, environmental, and climate conditions quantitation should be improvised [40].

## 6. Conclusions

This study proposes a new framework for the fatigue crack growth rate in aluminum alloys usually utilized in aircraft manufacturing, specifically 7055 T7511, 2324 T39, and 6013 T651. A comprehensive modeling and analysis was undertaken to accurately predict the fatigue crack growth in aluminum aircraft alloys. A hybrid DLFCO-DNN framework has been proposed to utilize experimental data for training the predictive models. Existing models based on physical models lack robustness and accuracy demonstrated by the proposed model. The quantitative and statistical analysis made using statistical indices re-affirms the superior performance of DLFCO-DNN in terms of training, testing, and run time of the algorithms. Moreover, the accuracy in prediction as compared to recent works is up to 4–8 times higher; meanwhile, the DLFCO-DNN algorithm complexity allows for hardware implementation on less costly controllers such as c2000 for real-time functionality. In light of improvised results, it is safe to conclude that the proposed novel framework hybrid fatigue crack growth rate prediction is highly effective.

## Figures and Tables

**Table 1:** Brief summary of the literature work.

SN.	Reference	Technique(s)	Summary
1.	Hu. Dianyin et al. [13]	Gaussian Process Regression (GPR)	Bayesian-based calibration method is presented to improve computational efficacy and accuracy for FCG life prediction. In comparison with experimental data, predicted results are bounded within a factor of ±2.0
2.	Ma. Xinran et al. [14]	Increment Learning Scheme	A fully connected neural network is presented using the Increment Learning Scheme for crack growth under tension on the specimens of 7B04 aluminum and TA15 titanium alloys. The proposed method is superior to conventional fitting techniques. The minimum achieved MSE is 0.10 for aluminum alloy for R = 0.06
3.	Mortazavi et al. [15]	Radial Basis Function Neural Network (RBF-NN)	Radial basis function-based neural network approach is presented to predict FCG behavior. The proposed technique has been verified through experimentation on aluminum and titanium alloys. This model handles non-linearity in a better way for both long and short cracks
4.	Wang et al. [16]	Extreme Learning Machine, RBF-NN, and GA-BP	Three different machine learning algorithms (MLAs) are compared for FCG prediction. The algorithms are tested on datasets of different alloys of aluminum and titanium. MLAs are compared with each other and with the classical K* approach. ELM outperforms its counterparts with minimum MSE, i.e., 3.12 × 10^−8^
5.	Rovinelli et al. [17]	Bayesian Networks	This study utilizes in situ experimental and crystal plasticity simulations study multimodal data with Bayesian networks to predict the fatigue crack growth rate in 3D for small cracks. A non-local driving force is postulated to construct a data-driven probabilistic crack propagation framework.
6.	Nejad et al. [18]	Rockwell micro-hardness experiment	Prediction of fatigue crack propagation and fractography of rail steel is made using Machine Learning algorithms. The modified Paris model is used to estimate fatigue crack growth rates. Fatigue crack growth and hardness tests are carried out by fractography studies on fractured specimens. A three-dimensional boundary element method is used for fatigue crack growth study under a stress field.

**Table 2:** Parameter descriptions of model used for prediction.

Parameter	Value
Number of Layers	4
Number of Hidden Layers	2
Number of Neurons in Hidden Layer	6
Number of Weights and Biases	59
Number of Iterations	50
Activation Function	RBF
Cost Function	NRMSE
Training Algorithm	DLFCOA

**Table 3:** Test conditions of alloy hardware experiments in the lab.

Specimen Type	M (T)
Notch Lenght (2$a_{0}$)	10.2 mm
Width (W)	101.6 mm
Thickness (B)	2.2–3.1 mm (Variable)
Test Frequency	15 Hz
da/dN	$1\times 10^{-12}-1\times 10^{-4}$ m/cycle
Orientation	L-T
Lab Air Temp.	24 °C
Relative Humidity (R.H)	50–55%

**Table 4:** Alloy 7055 T7511 algorithm performance results.

Tech	Testing MSE	Training MSE	Run Time (s)
DLFCO-DNN	1.33 × 10^−10^	5.394 × 10^−11^	98.43 ms
MFA-DNN	4.55 × 10^−10^	9.103 × 10^−11^	101.45 ms
GWO-DNN	8.21 × 10^−10^	8.547 × 10^−10^	108.21 ms
PSO-DNN	1.67 × 10^−9^	2.409 × 10^−10^	112.04 ms

**Table 5:** Alloy 7055 T7511 statistical analysis results.

Tech	MAE	RMSE	*R* ^2^	Mean RE
DLFCO-DNN	7.11 × 10^−5^	9.34 × 10^−8^	0.1213	0.0512
MFA-DNN	2.98 × 10^−4^	7.97 × 10^−8^	0.1976	0.9563
GWO-DNN	3.09 × 10^−5^	2.65 × 10^−6^	0.2015	1.1453
PSO-DNN	3.85 × 10^−4^	1.01 × 10^−7^	0.2768	1.4300

**Table 6:** Alloy 6013 T651 algorithm performance results.

Tech	Testing MSE	Training MSE	Run Time (s)
DLFCO-DNN	1.01 × 10^−10^	2.44 × 10^−11^	99.01 ms
MFA-DNN	2.44 × 10^−10^	8.01 × 10^−11^	102.30 ms
GWO-DNN	7.01 × 10^−10^	4.40 × 10^−10^	109.22 ms
PSO-DNN	4.21 × 10^−9^	1.99 × 10^−10^	114.23 ms

**Table 7:** Alloy 6013 T651 statistical analysis results.

Tech	MAE	RMSE	*R* ^2^	Mean RE
DLFCO-DNN	7.14 × 10^−5^	7.33 × 10^−6^	3.48 × 10^−4^	0.0141
MFA-DNN	4.26 × 10^−4^	1.21 × 10^−4^	4.22 × 10^−4^	0.2114
GWO-DNN	5.65 × 10^−4^	5.85 × 10^−5^	5.04 × 10^−4^	0.5468
PSO-DNN	9.23 × 10^−4^	1.99 × 10^−4^	5.47 × 10^−4^	0.5090

**Table 8:** Alloy 2324 T39 results.

Tech	Testing MSE	Training MSE	Run Time (s)
DLFCO-DNN	2.71 × 10^−10^	5.04 × 10^−11^	96.01 ms
MFA-DNN	3.97 × 10^−10^	1.21 × 10^−10^	106.30 ms
GWO-DNN	6.05 × 10^−9^	5.43 × 10^−10^	112.24 ms
PSO-DNN	9.01 × 10^−8^	1.99 × 10^−9^	115.03 ms

**Table 9:** Alloy 2324 39 results.

Tech	MAE	RMSE	*R* ^2^	Mean RE
DLFCO-DNN	1.0913 × 10^−4^	1.0971 × 10^−5^	0.9521	0.0911
MFA-DNN	8.4101 × 10^−4^	8.4721 × 10^−5^	0.9781	0.4023
GWO-DNN	1.7400 × 10^−4^	1.6021 × 10^−5^	0.9901	0.2581
PSO-DNN	8.6701 × 10^−4^	8.4431 × 10^−5^	0.9921	0.4221

## Data Availability

Not applicable.
